# Neoadjuvant palbociclib in women with operable, hormone receptor-positive breast cancer

**DOI:** 10.1530/ERC-24-0353

**Published:** 2025-09-11

**Authors:** Takayuki Ueno, Louis W C Chow, Wonshik Han, Chiun Sheng Huang, G Bruce Mann, Satoshi Morita, Hironori Haga, Elham Fakhrejahani, Takayuki Kobayashi, Hiroko Bando, Kenichi Inoue, Mariko Tokiwa, Hirofumi Suwa, Tomoyuki Aruga, Sachiko Minamiguchi, Yosuke Yamada, Yuko Tanabe, Masahiro Takada, Toshinari Yamashita, Hiroji Iwata, Chi-Feng Chung, Sachiko Takahara, Eriko Tokunaga, Shigeru Imoto, Eun Sook Lee, Yasuaki Sagara, Jee Hyun Kim, Richard H DeBoer, Hyun-Ah Kim, Hung Wen Lai, Ming Feng Hou, Michelle White, Yoshiko Umeyama, Masakazu Toi

**Affiliations:** ^1^ Breast Surgical Oncology, Breast Oncology Center, Cancer Institute Hospital, Japanese Foundation for Cancer Research (JFCR), Tokyo, Japan; ^2^ UNIMED Medical Institute Comprehensive Center for Breast Diseases, Hong Kong, Hong Kong; ^3^ Seoul National University Hospital, Seoul, Korea; ^4^ National Taiwan University Hospital, Taipei, Taiwan; ^5^ The Royal Melbourne Hospital, Melbourne, Australia; ^6^ Department of Biomedical Statistics and Bioinformatics, Kyoto University Graduate School of Medicine, Kyoto, Japan; ^7^ Department of Diagnostic Pathology, Kyoto University Hospital, Kyoto, Japan; ^8^ Kyoto Breast Cancer Research Network, Kyoto, Japan; ^9^ Department of Breast Medical Oncology, The Cancer Institute Hospital of JFCR, Tokyo, Japan; ^10^ University of Tsukuba Hospital, Tsukuba, Japan; ^11^ Division of Breast Oncology, Saitama Cancer Center, Saitama, Japan; ^12^ Kobe City Medical Center General Hospital, Kobe, Japan; ^13^ Hyogo Prefectural Amagasaki General Medical Center, Amagasaki, Japan; ^14^ Tokyo Metropolitan Cancer and Infectious Disease Center Komagome Hospital, Bunkyo, Japan; ^15^ Toranomon Hospital, Minato, Japan; ^16^ Department of Breast Surgery, Kyoto University Graduate School of Medicine, Kyoto, Japan; ^17^ Kanagawa Cancer Center, Yokohama, Japan; ^18^ Aichi Cancer Center, Nagoya, Japan; ^19^ Koo Foundation Sun Yat-Sen Cancer Center, Taipei City, Taiwan; ^20^ Medical Research Institute, Kitano Hospital, Osaka, Japan; ^21^ National Hospital Organization Kyushu Cancer Center, Fukuoka City, Japan; ^22^ Kyorin University Hospital, Mitaka, Japan; ^23^ National Cancer Center, Goyang, Korea; ^24^ Hakuaikai Sagara Hospital, Kagoshima, Japan; ^25^ Department of Internal Medicine, Seoul National University Bundang Hospital, Seoul National University College of Medicine, Seongnam-si, Korea; ^26^ Peter MacCallum Cancer Centre, Melbourne, Australia; ^27^ Korea Cancer Center Hospital, Nowon-gu, Korea; ^28^ Changhua Christian Hospital, Changhua City, Taiwan; ^29^ Kaohsiung Medical University Hospital, Kaohsiung City, Taiwan; ^30^ Monash Medical Centre, Clayton, Australia; ^31^ Pfizer R&D Japan, Shibuya, Japan

**Keywords:** breast cancer, neoadjuvant endocrine therapy, palbociclib, CDK4/6, pre-operative endocrine prognostic index (PEPI)

## Abstract

The addition of a cyclin-dependent kinase 4/6 (CDK4/6) inhibitor to endocrine therapy augments biological response in breast cancer. This phase III randomized, double-blind study evaluated the efficacy of adding palbociclib to neoadjuvant endocrine therapy (NET) for operable, hormone receptor-positive human epidermal growth factor receptor 2 (HER2)-negative breast cancer. Patients randomly received 16 weeks of endocrine therapy (letrozole for postmenopausal and tamoxifen plus ovarian function suppression for pre-/perimenopausal patients) plus palbociclib or placebo. The co-primary endpoints included preoperative endocrine prognostic index (PEPI) score and EndoPredict (EPclin) risk score according to the gatekeeping procedure. Of 141 randomized patients, 130 completed the treatment with surgical samples evaluable for endpoints in 126 patients. The proportion of patients with a low, moderate, and high PEPI score was 15.2, 50.0, and 34.8% in the palbociclib arm and 13.3, 55.0, and 31.7% in the placebo arm, respectively, with no statistical difference (one-sided *P* = 0.563). Statistical analysis was not performed on EPclin risk score. No new safety signals were reported. Permanent treatment discontinuation by adverse events was reported for seven (9.7%) and zero patients in the palbociclib and placebo arms, respectively. In conclusion, the addition of palbociclib to NET did not improve the efficacy. ClinicalTrials.gov NCT03969121.

## Background

In hormone receptor (HR)-positive breast cancer, adjuvant endocrine therapy has been shown to reduce the recurrence risk substantially and to prolong survival (1, 2). Neoadjuvant endocrine therapy (NET) has been employed to increase the breast conservation rate and to improve surgical outcomes (3, 4, 5, 6, 7, 8). NET further provides an *in vivo* analysis of sensitivity or resistance to endocrine treatment (9).

The association between response to NET and survival has been studied. Poor clinical response to NET has been shown to indicate poor survival (10). Early biological response to endocrine therapy indicated by on-treatment or post-treatment Ki-67 labeling index (LI) has been demonstrated to predict long-term outcomes (11, 12). Based on these notions, preoperative endocrine prognostic index (PEPI) scores, generated using post-treatment Ki-67 and estrogen receptor (ER) status together with post-treatment tumor burden, have been shown to predict patient outcomes (13, 14).

Incorporation of multigene assays into the decision-making of treatment with NET has been examined. Recurrence score (RS) has been suggested to associate with clinical response to NET and neoadjuvant chemotherapy (NAC) (15, 16, 17, 18, 19). The WSG-ADAPT-HR+/human epidermal growth factor receptor (HER)2– trial integrated RS and early biological response into treatment decision-making to guide systemic treatment and suggested that patients with intermediate RS with good biological response to NET could safely omit chemotherapy even with up to three involved lymph nodes (20). The CORALLEEN trial, in which letrozole plus ribociclib was compared with chemotherapy in the neoadjuvant setting for luminal B breast cancer, utilized PAM50 risk of recurrence score, which integrates tumor gene expression data, tumor size, and nodal status to predict prognosis, as the primary endpoint and showed molecular downstaging in some patients with luminal B breast cancer who received letrozole plus ribociclib (21). Recently, EndoPredict, a multigene assay that predicts the risk of distant recurrence in patients with operable ER+ HER2– breast cancer, has also been shown to be predictive of response to NET and NAC (22, 23). In addition, using samples from the TransATAC study, the EPclin risk score, which integrates EndoPredict with nodal status and tumor size, has been suggested to provide more prognostic information than RS in patients with ER+ HER2– breast cancer (24).

Cyclin-dependent kinase 4/6 (CDK4/6) inhibitors added to endocrine therapies have been shown to prolong progression-free survival in advanced breast cancer (25). In the neoadjuvant setting, a single-arm study with letrozole and palbociclib was conducted (26). Patients were given letrozole and palbociclib for 16 weeks, and the clinical response rate was 85%. This study also showed that EndoPredict scores as well as Ki-67 were significantly reduced after treatment. A phase II randomized study, the PALLET trial, evaluated the efficacy of palbociclib in addition to neoadjuvant letrozole, and showed that adding palbociclib to letrozole significantly enhanced the suppression of proliferation indicated by Ki-67 without an increased clinical response rate over 14 weeks in postmenopausal patients (27). With this background, a phase III study was conducted to evaluate the efficacy of palbociclib added to NET in pre-/peri- and postmenopausal patients with operable HR+ HER2– breast cancer.

## Patients and methods

### Clinical trial design

This was a phase III multicenter, international, randomized, double-blind study that compared NET plus palbociclib with NET plus placebo in untreated pre-/peri- and postmenopausal women with operable HR+ (ER and/or progesterone receptor +), HER2– breast cancer. HR+ was defined as HR expression ≥1% in this study. The other major eligibility criteria included tumor size ≥15 mm, T1c-3 N0-1, Ki-67 LI ≥ 14% by central assessment, and no previous history of radiotherapy or systemic therapy for breast cancer. The assessment of Ki-67 was carried out as follows: one pathologist identified a hot spot (tumor area with a high LI) under low-power magnification and calculated the Ki-67 positivity rate among 500 tumor cells. The second pathologist reviewed the same specimen to verify the result. If both assessments classified the rate as either below 20% or 30% or higher, the initial report was accepted. If there was a discrepancy, both pathologists reviewed the specimen together using a multi-head microscope and reached a consensus. The exclusion criteria included male, multicentric breast cancer, previous use of selective ER modulators, any CDK4/6 inhibitor, everolimus, or any agent that inhibits the phosphatidylinositol-3 kinase (PI3K)-mammalian target of rapamycin (mTOR) pathway as a mechanism of action, and prior history of other malignancy within 5 years aside from basal cell carcinoma of the skin or carcinoma *in situ* of the uterine cervix.

Eligible patients were randomly assigned 1:1 to receive 16 weeks of endocrine therapy plus palbociclib (125 mg/day at a 3-weeks-on/1-week-off schedule) or endocrine therapy plus placebo. Endocrine therapy included letrozole (2.5 mg per day) in postmenopausal patients and tamoxifen (20 mg per day) plus ovarian function suppression by either leuprorelin or goserelin in pre- and perimenopausal patients. Randomization was stratified by menopausal status (pre-/peri- vs post-), Ki-67 LI (<20% vs ≥ 20%), and nodal status (positive vs negative). Surgery was planned after the completion of neoadjuvant treatment. Palbociclib or placebo was stopped 2–3 weeks before surgery, and endocrine therapy could be continued until 1 day before surgery. Patients were withdrawn from the study treatment in the case of disease progression, unacceptable toxicities, or withdrawal of patient consent.

The protocol was approved by the ethics committee of each institute (#2018-0015 approved by the ethics committee of the Cancer Institute Hospital of JFCR), and written informed consent was obtained from all participating patients.

### Outcomes

The co-primary endpoints included PEPI score based on relapse-free survival and EPclin risk score. Secondary endpoints included clinical response rate, drop in Ki-67 ≤ 2.7%, pathological complete response (pCR) rate, breast-conserving rate, adverse events, and biomarker studies. Ki-67 LI and EndoPredict-related measurements were centrally assessed. Clinical response was evaluated according to the Response Evaluation Criteria in Solid Tumors version 1.1. Safety and graded adverse events were evaluated according to Common Terminology Criteria for Adverse Events version 4.03.

### Statistical analysis

The co-primary endpoints were sequentially analyzed on a modified intent-to-treat (mITT) basis according to the gatekeeping procedure (28) to strictly control the family-wise type I error rate at a two-sided alpha level of 0.05. We first analyzed the PEPI score, and if statistical significance was detected at a significance level *α* = 0.05, the statistical significance of EPclin risk score would be assessed at a significance level *α* = 0.05. The primary population for analysis of the primary endpoint was an mITT population defined as all patients randomized excluding patients with missing tumor samples, missing data for PEPI or EPclin risk score, or withdrawal of consent for use of all data. Patients with HER2+ at surgery were excluded from the mITT population, as EPclin risk score was developed for patients with HR+ HER2– breast cancer. In the case of pCR, PEPI and EPclin risk score were considered as zero. All patients who received at least one dose of study treatment were evaluable for safety assessment.

The relative dose intensity was calculated as follows: actual overall dose intensity/intended dose intensity. The actual overall dose intensity was calculated as follows: sum over all cycles of the actual total dose per cycle/sum over all cycles of the actual number of weeks in the cycle. The intended dose intensity was calculated as follows: intended total dose per cycle/intended number of weeks in the cycle.

The target sample size was 100 patients in each arm, which was calculated with <5% type I error rate (two-sided) and 80% power based on the following hypotheses on PEPI score: moving 15% of patients with high-risk to the moderate-risk group; moving 5% of patients with high-risk to the low-risk group; moving 5% of patients with moderate-risk to the low-risk group. The comparisons in the PEPI score were conducted using the Wilcoxon test to compare the frequencies in shifts across the risk categories (‘low’, ‘med’, ‘high’) between palbociclib treatment and placebo control arms. Chi-square test with continuity adjustment was used to compare the EPclin risk score in terms of the proportions of patients with ‘high’ risk between arms. As ad hoc analysis, a subgroup analysis for PEPI and an analysis of time to response were conducted. A subgroup analysis for PEPI was performed using the Wilcoxon test. The time-to-response curve was drawn by the hazard plot, and the comparison was conducted using the log-rank test. Hazard ratio was calculated by the Cox proportional hazards model. All analyses were performed using SAS^®^ Version 9.4 (SAS Institute Japan Ltd, Japan).

## Results

**Figure 1:**
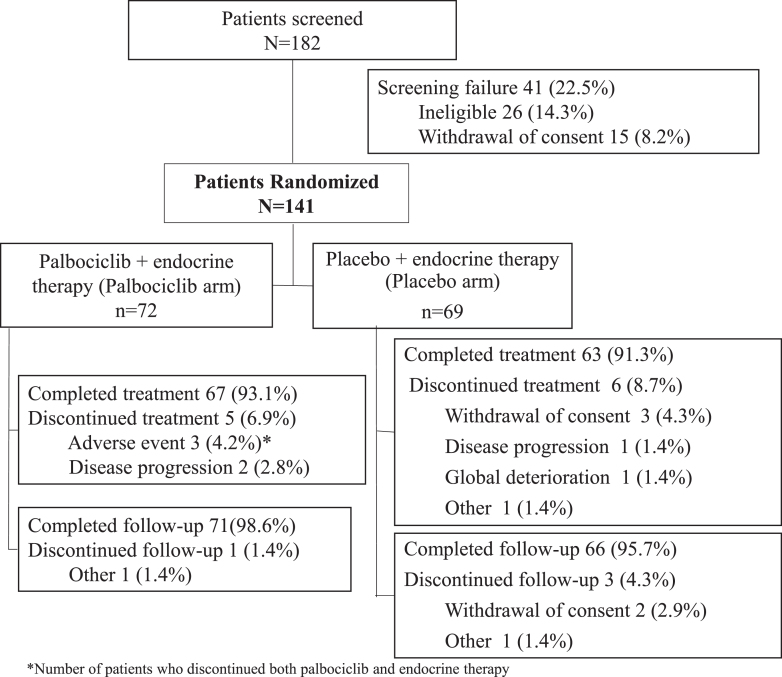
Study flow diagram.

Between 16 July 2019 and 7 July 2021, 141 eligible patients were randomized from 25 participating institutes in Korea, Taiwan, Hong Kong, Australia, and Japan: 72 patients in the palbociclib arm and 69 in the placebo arm. One hundred and thirty patients completed the treatment, and surgical samples were collected to evaluate endpoints. Randomization was well-balanced in terms of age, menopausal status, and cancer stage (Supplementary Table 1 (see section on Supplementary materials given at the end of the article)). Five patients in the palbociclib arm and six patients in the placebo arm discontinued treatment mainly due to adverse events, withdrawal of consent, or disease progression (Fig. 1). Six patients in the palbociclib arm and nine patients in the placebo arm were excluded from the mITT population due to missing tumor samples, missing data for PEPI or EPclin risk score, or withdrawal of consent. The background characteristics in the mITT population were well-balanced between arms (Table 1). The median duration of palbociclib and placebo treatment was 15.21 and 15.00 weeks, respectively. Treatment with palbociclib/placebo was initiated in four cycles for 88.9% patients in the palbociclib arm and 92.8% in the placebo arm. The median relative dose intensity of palbociclib and placebo was 81.53 and 99.12%, respectively.

**Table 1:** Background characteristics of mITT population.

	Category	Palbociclib + endocrine therapy	Placebo + endocrine therapy
		(*n* = 66) (%)	(*n* = 60) (%)
Age (years)	Median (range)	57 (38–84)	54 (29–84)
Menopausal status	Pre/peri-menopausal	29 (43.9)	25 (41.7)
	Post-menopausal	37 (56.1)	35 (58.3)
T	T1	5 (7.6)	4 (6.7)
	T2	57 (86.4)	56 (93.3)
	T3	4 (6.1)	0 (0.0)
N	N0	51 (77.3)	45 (75.0)
	N1	15 (22.7)	15 (25.0)
Stage	I	5 (7.6)	4 (6.7)
	IIA	42 (63.6)	41 (68.3)
	IIB	19 (28.8)	15 (25.0)
ER	Positive	66 (100.0)	60 (100.0)
	Negative	0 (0)	0 (0)
PgR	Positive	64 (97.0)	55 (91.7)
	Negative	2 (3.0)	5 (8.3)
Histopathological grade	Grade 1	6 (9.1)	8 (13.3)
	Grade 2	49 (74.2)	36 (60.0)
	Grade 3	10 (15.2)	15 (25.0)
	Unknown	1 (1.5)	1 (1.7)
Ki-67 labeling index	<20%	11 (16.7)	10 (16.7)
	≥ 20%	55 (83.3)	50 (83.3)
	<30%	38 (57.6)	42 (70.0)
	≥ 30%	28 (42.4)	18 (30.0)
			
Endocrine therapy	Tamoxifen	29 (43.9)	25 (41.7)
	Letrozole	37 (56.1)	35 (58.3)

### Biological response

The proportion of patients who had a low, moderate, and high PEPI score was 15.2, 50.0, and 34.8% in the palbociclib arm, respectively, and 13.3, 55.0, and 31.7% in the placebo arm, respectively (Table 2). There was no statistically significant difference in PEPI score between the two arms (one-sided *P*-value = 0.563). Thus, the statistical analysis for EPclin risk score was not performed. The result of EPclin risk score is shown in Table 3. The proportion of patients who had a high-risk EPclin risk score was numerically lower in the palbociclib arm than in the placebo arm (62.1 vs 68.3%), although statistical testing was not performed on EPclin risk score because of the gatekeeping procedure. Exploratory subgroup analyses for PEPI were performed, and no subgroup showed a difference in PEPI between the two treatment arms (Supplementary Table 2).

**Table 2:** PEPI score (mITT population).

	Palbociclib arm (*n* = 66)		Placebo arm (*n* = 60)		
	*n*	%	*n*	%	*P*-value
Low	10	15.2	8	13.3	0.563
Moderate	33	50	33	55	
High	23	34.8	19	31.7	

**Table 3:** EPclin score (mITT population).

	Palbociclib arm (*n* = 66)		Placebo arm (*n* = 60)	
	*n*	%	*n*	%
Low	25	37.9	19	31.7
High	41	62.1	41	68.3

The rate of Ki-67 ≤ 2.7% at surgery was 20.0% for palbociclib and 23.3% for placebo, which was not statistically different. The mean Ki-67 level at each time point during treatment is shown in Table 4. It was reduced from baseline to cycle 1 day 15 in both palbociclib and placebo arms; and partially recovered at the time of surgery.

**Table 4:** Ki-67 labeling index during treatment (mITT population).

	Palbociclib			Placebo		
	*n*	Mean	SD	*n*	Mean	SD
Baseline	66	29.18	12.61	60	28.21	11.55
C1D15	20	1.92	2.95	13	7.52	6.75
At surgery	65	12.68	14.62	60	11.27	10.52

In the ITT population, one patient (1.5%) showed pCR in the palbociclib arm, and no pCR was observed in the placebo arm.

### Clinical response

The clinical response rate assessed by ultrasound in the intent-to-treat (ITT) population is shown in Table 5. There was no significant difference in the response rate between palbociclib (55.6%) and placebo (44.9%) treatment arms. In an exploratory analysis, time to ultrasound response was analyzed in the ITT population (Supplementary Fig. 1). There was no difference between the two treatment arms (*P* = 0.484).

**Table 5:** Clinical response by ultrasound (ITT population).

Treatment	CR	PR	SD	PD	NE	CRR
Palbociclib (*n* = 72)	1	39	31	0	1	55.6%
Placebo (*n* = 69)	0	31	37	1	0	44.9%

The breast conservation rate showed no significant difference between the palbociclib arm and the placebo arm (57.4 vs 67.2%) in the ITT population.

### Safety

No new safety signals were found in the study (Table 6). The most commonly reported adverse events (≥20% of patients) in the palbociclib arm were neutropenia (87.5%), leukopenia (69.4%), stomatitis (33.3%), and anemia (22.2%). Procedural pain and nausea were observed similarly in both arms. Permanent discontinuation from the study in association with adverse events was reported for seven (9.7%) patients in the palbociclib arm and for zero patients in the placebo arm.

**Table 6:** Common adverse events reported by ≥ 20% of patients in either arm (any grade) (as treated population).

	Palbociclib arm (*n* = 72)		Placebo arm (*n* = 69)	
	*n*	%	*n*	%
Any treatment emergent adverse event	71	98.6	66	95.7
Neutropenia	63	87.5	3	4.3
Leukopenia	50	69.4	1	1.4
Procedural pain	28	38.9	33	47.8
Stomatitis	24	33.3	6	8.7
Anemia	16	22.2	1	1.4
Nausea	10	13.9	14	20.3

## Discussion

This study did not meet its primary objective of improved efficacy measured by PEPI score and EPclin risk score. No statistically significant differences of PEPI score were observed between the two treatment arms. In addition, the combination of palbociclib with neoadjuvant endocrine treatment did not show additional significant therapeutic effects to placebo with endocrine therapy in secondary endpoints including clinical response rate, reduction in Ki-67 level, pCR, and breast-conserving rate. Our results are in accordance with a recently published phase III randomized trial, SAFIA trial, in which neoadjuvant fulvestrant with or without palbociclib was given in patients with low-risk ER+ HER2– breast cancer, and no difference in pCR rate was observed (29). In addition, our results are consistent with the results of adjuvant palbociclib trials, PALLAS and Penelope-B, in which the addition of palbociclib did not show survival benefit in the adjuvant settings, although the patient backgrounds were different (30, 31).

There have been several studies that examined an addition of a CDK4/6 inhibitor to an endocrine therapy in the neoadjuvant settings. MONALEESA-1, neoMONARCH, and FELINE studies examined an addition of a CDK4/6 inhibitor including ribociclib and abemaciclib compared to an endocrine therapy alone preoperatively. All three studies demonstrated that an addition of a CDK4/6 inhibitor showed a greater suppression of Ki-67 at 14 days than an endocrine therapy alone (https://clinicaltrials.gov/study/NCT02712723; 32, 33). However, FELINE study showed no difference in Ki-67 suppression at surgery (https://clinicaltrials.gov/study/NCT02712723). Our study showed a trend toward a greater suppression of Ki-67 at 14 days in the palbociclib arm but no difference in Ki-67 suppression at surgery between arms, which is consistent with FELINE study. PALLET trial examined an addition of palbociclib to letrozole and showed a greater decrease in Ki-67 in arms containing palbociclib at the end of the treatment (27). This is in contrast with our study and may be explained, to some extent, by the difference in the timing of palbociclib cessation before surgery and the method of tissue sampling as described below. Indeed, the aforementioned CORALLEEN trial, in which surgery was carried out within 7 days after the last dose of ribociclib, showed that letrozole plus ribociclib gave the mean post-treatment Ki-67 of 8.4%, which is lower than that in our study (21). NeoPAL study, in which letrozole plus palbociclib was compared to chemotherapy in the neoadjuvant setting, suggested that the combination of letrozole and palbociclib gave the mean post-treatment Ki-67 of 1.17% in post-treatment samples at the surgery that was performed 24 h after the last dose of palbociclib and might allow sparing chemotherapy in some patients with high-risk luminal breast cancer (34, 35). Thus, the timing of surgery after the last dose of palbociclib (2 to 3 weeks) in this study may have contributed, to some extent, to the negative result of the study.

No new safety signals were observed in this study. Myelosuppression including neutropenia, leukopenia, and anemia was observed more frequently in the palbociclib arm than in the placebo arm, which is in agreement with the previous studies (29, 30, 31). An ad hoc analysis in which an association between treatment efficacy and alterations in blood indicators including neutrophil count, hemoglobin, and platelet count in the combination therapy group was performed and showed no association between them (data not shown).

Rebound in cell proliferation after the cessation of a CDK4/6 inhibitor has been reported. In the NeoPalAna trial, in which palbociclib was given in addition to anastrozole until 3 to 5 weeks before surgery, Ki-67 rebound was observed with higher levels of Ki-67 at surgery than at cycle 1 day 15, while the rebound disappeared when additional palbociclib was given until surgery (36). In the phase II neoMONARCH trial, in which abemaciclib was added to anastrozole, when study treatment was stopped more than 4 days before the final biopsy, the Ki-67 rebounded (Ki-67 > 2.7%) in 69% of the tumors compared with 11% for patients who remained on study treatment and 32% for patients who had stopped for one to 4 days before biopsy (33). In this study, Ki-67 levels were drastically reduced at cycle 1 day 15 in both arms with more pronounced reduction in the palbociclib arm and rebounded at the time of surgery to similar levels in both arms (Table 4). This study stopped palbociclib 2 to 3 weeks before surgery, which would have resulted in a rebound increase in cell proliferation because we considered that Ki-67 after rebound might be more predictive for survival because it was considered a more stable state after CDK4/6 inhibitor cessation. This rebound may affect the analysis of endpoints including Ki-67, PEPI, and EPclin risk score. In the PALLET trial, core biopsy was to be taken within 48 h of the last dose of trial treatment, which may have, to some extent, contributed to the discrepancy from our study (27).

The most appropriate endpoint for NET plus a CDK4/6 inhibitor remains uncertain. NeoMONARCH trial used the change in Ki-67 expression from baseline to 2 weeks of therapy as primary endpoint and showed a larger decrease of Ki-67 in the abemaciclib arm than in the anastrozole arm: a similar trend was observed in this study. PEPI score was used as a co-primary endpoint in this study because it is one of the most widely used endpoints for NET (37). PEPI score has been shown to be associated with long-term survival after NET and is also used as the primary endpoint in the FELINE study (https://clinicaltrials.gov/study/NCT02712723). However, it has not been shown whether PEPI score is indicative of survival after NET plus a CDK4/6 inhibitor. Especially considering the rebound in Ki-67 after cessation of a CDK4/6 inhibitor, it is unclear whether post-treatment Ki-67, which is a component of the PEPI score, is associated with survival and, if so, whether Ki-67 expression before or after rebound is more predictive for survival. It is of critical importance to study long-term survival in association with Ki-67 LI and PEPI score after NET plus a CDK4/6 inhibitor.

There are a number of limitations in this study. First, the recruitment of patients was slow, in part due to COVID-19 pandemic, and the planned enrollment could not be achieved due to the time limitation. Thus, the statistical power was decreased. Second, we used PEPI and EPclin scores as co-primary endpoints but endpoints, for NET plus a CDK4/6 inhibitor have not been definitively established as already discussed. In addition, because of the nature of the endpoints, not ITT population but mITT population was used for the assessment of the endpoints, which reduced the sample size, although the sample size was calculated based on the mITT population. Third, rebound in cancer cell proliferation after cessation of a CDK4/6 inhibitor is recognized, and the prognostic relevance of the rebound is unknown. Fourth, the assessment of Ki-67 at the end of treatment was performed with surgical specimens but not with core biopsy samples in this study. This may lead to delayed fixation compared with core biopsy, which, in general, allows quick fixation after sample collection and may additionally explain the difference from PALLET trial, in which core biopsy was taken within 48 h after the last dose of trial treatment.

In conclusion, the addition of palbociclib to NET did not improve efficacy measured by PEPI score in patients with operable HR+ HER2– breast cancer. Further analyses including biomarker assessments are ongoing. The role of chemotherapy after neoadjuvant therapy is under investigation.

## Supplementary materials



## Declaration of interest

Takayuki Ueno received lecture fees from Astra Zeneca, Chugai Pharmaceutical, Eisai Co. Ltd and Novartis Pharma KK. Louis W C Chow received funding or grants from Merck & Co Inc, Novartis Pharmaceuticals Corporation, and OBI Pharma Inc. Wonshik Han reports equity and stock ownership of DCGen Inc, and received advisory fees, funding, grants, and lecture fees from Takeda, Shinpoong, Kwangdong, Agendia, Handok, Hanall, Novartis, Celltrion, Hanlim, Genomic Health, Kyowa Kirin, Pfizer, Roche, Alvogen, and Amgen. Chiun Sheng Huang received advisory fees, funding, grants, and lecture fees from AstraZeneca, Daiichi Sankyo, EirGenix, Lilly, MSD, Novartis, OBI Pharma, Pfizer, Roche, Seagen, Gilead Sciences Inc, and Aston Sci. Satoshi Morita received lecture fees from Astellas Pharma Inc, AstraZeneca Kabushiki Kaisha, Bristol Myers Squibb Co, Chugai Pharmaceutical Co Ltd, Eisai Co Ltd, Eli Lilly Japan KK, MSD KK, Novartis Pharma Japan, Ono Pharmaceutical Co Ltd, Pfizer Japan Inc, and Taiho Pharmaceutical Co Ltd. Hiroko Bando received advisory and lecture fees from AstraZeneca, Chugai Pharmaceutical Co Ltd, Daiichi Sankyo Inc, Eisai Co Ltd, Eli Lilly, Kyowa Kirin Inc, Novartis, and Pfizer Japan Inc. Kenichi Inoue received funding and grants from AstraZeneca, Chugai Pharma, Daiichi Sankyo, MSD, Ono, Pfizer, and Takeda. Tomoyuki Aruga received lecture fees from AstraZeneca, Chugai, Eli Lilly, and Pfizer. Yuko Tanabe received funding and grants from Daiichi Sankyo, MSD, and Taiho. Masahiro Takada received lecture fees, funding, and grants from AstraZeneca, Chugai, Daiichi Sankyo, Eisai, Lilly, Medbis, Pfizer, and Yakult. Toshinari Yamashita received lecture fees, funding, and grants from Chugai, Daiichi Sankyo, Eisai, Eli Lilly, AstraZeneca, Taiho, Pfizer, MSD, Novartis Pharma, Kyowa Kirin, Gilead Sciences, Ono, Seagen, and Nippon Kayaku. Hiroji Iwata received funding, grants, and advisory and lecture fees from Amgen, AstraZeneca, Chugai, Daiichi Sankyo, Lilly, MSD, Novarti, Pfizer, and Sanofi. Chi-Feng Chung received advisory fees from AstraZeneca, CANbridge Pharma, Daiichi Sankyo, EirGenix, Lotus, and Roche. Eriko Tokunaga received lecture fees from AstraZeneca, Chugai, Daiichi Sankyo, Eisai, Eli Lilly, Kyowa Kirin, Nihon Kayaku, and Pfizer. Shigeru Imoto received funding and grants from Chugai, Eisai, and Taiho Pharma. Yasuaki Sagara received lecture fees from AstraZeneca, Chugai, Daiichi Sankyo, Eisai, Eli Lilly, Kyowa Hakko Kirin, MSD, and Pfizer. Jee Hyun Kim received advisory and lecture fees from Bixink, Daiichi Sankyo, Eisai, Everest Medicines, Lilly Korea, Novartis, Ono Pharma Korea Ltd, Pfizer Korea, Roche Diagnostics, Roche Korea, Sanofi-Aventis, and Yuhan. Richard H DeBoer received lecture and advisory fees, funding, and grants from Amgen Australia, AstraZeneca, Eli Lilly, Gilead Australia, MSD, and Pfizer. Michelle White received advisory fees from Pfizer. Yoshiko Umeyama is an employee of Pfizer R&D Japan and holds stock in Pfizer Inc. Masakazu Toi received funding, grants, advisory, and lecture fees from AFI Technologies, Japan Breast Cancer Research Group, Kyoto Breast Cancer Research Network, Astellas, AstraZeneca, Athenex Oncology, Bertis, BMS, Chugai, Daiichi Sankyo, Devicore Medical Japan, Eisai, Eli Lilly, Exact Sciences, GL Sciences, Kansai Medical Net, Luxonus, MSD, Nippon Kayaku, Novartis, Pfizer, Sanwa Shurui, Shimadzu, Shionogi, Taiho, Takeda, Terumo, and Yakult. A part of the findings was presented at San Antonio Breast Cancer Symposium 2022 under the title ‘Neoadjuvant hormonal therapy plus palbociclib versus hormonal therapy plus placebo in women with operable, hormone sensitive and HER2-negative primary breast cancer’ (38).

## Funding

This study was funded by Pfizer Inc.

## Author contribution statement

TU, LWC, SM, and MT were responsible for conceptualization. SM and EF handled data curation. SM performed the formal analysis. MT helped in funding acquisition. TU, LWC, WH, CSH, GBM, TK, HB, KI, MT, HS, TA, YT, MT, TY, HI, CFC, ST, ET, SI, ESL, YS, JHK, RHD, HAK, HWL, MFH, MW, and MT helped in investigation. TU, LWC, HH, SM, YY, YU, and MT contributed to methodology. EF managed the project administration. LWC and MT helped in supervision. All authors prepared the original draft. All authors reviewed and edited the manuscript.

## Data availability

Data associated with this study have not been deposited into a publicly available repository, and data will be made available upon reasonable request.
