# Supplementary material for: Neoadjuvant palbociclib in women with operable, hormone receptor-positive breast cancer
Source: Endocr Relat Cancer. 2025 Sep 11;32(9):e240353. doi: 10.1530/ERC-24-0353 (PMC12433830; doi:10.1530/ERC-24-0353)
Supplement: Supplementary file 1 [file supplementary_materials.pdf]

Supplementary Table 1 Background characteristics (ITT population)

	Category	Palbociclib + endocrine therapy (N=72)	Placebo+ endocrine therapy (N=69)
Age (years)	Median (range)	55.5 (38-84)	53 (29-84)
Menopausal status	Pre/peri-menopausal	31 (43.1)	30 (43.5)
	Post-menopausal	41 (56.9)	39 (56.5)
T	T1	5 (6.9)	5 (7.2)
	T2	62 (86.1)	63 (91.3)
	T3	5 (6.9)	1 (1.4)
N	N0	54 (75.0)	52 (75.4)
	N1	18 (25.0)	17 (24.6)
Stage	I	5 (6.9)	5 (7.2)
	IIA	44 (61.1)	46 (66.7)
	IIB	23 (31.9)	18 (26.1)
ER	Positive	72 (100.0)	69 (100.0)
	Negative	0 (0)	0 (0)
PgR	Positive	68 (94.4)	64 (92.8)
	Negative	4 (5.6)	5 (7.2)
Histopathological grade	Grade 1	7 (9.7)	9 (13.0)
	Grade 2	53 (73.6)	40 (58.0)
	Grade 3	11 (15.3)	19 (27.5)
	Unknown	1 (1.4)	1 (1.4)
Ki67 labelling index	< 20%	11 (15.3)	10 (14.5)
	>= 20%	61 (84.7)	59 (85.5)

	< 30%	40 (55.6)	45 (65.2)
	>= 30%	32 (44.4)	24 (34.8)
Endocrine therapy	tamoxifen	31(43.1)	29 (42.0)
	letrozole	41 (56.9)	40 (58.0)

( ): %

Supplementary Table 2 Subgroup analysis for PEPI between two treatment arms (mITT population)

	Category	p-value[1]
Menopausal status	Pre/peri-menopausal	0.169
	Post-menopausal	0.794
Stage	I	0.251
	IIA	0.366
	IIB	0.797
Histopathological grade	Grade 1	0.220
	Grade 2	0.372
	Grade 3	0.500
Ki67 labelling index	< 20%	0.970
	>= 20%	0.299
	< 30%	0.179
	>= 30%	0.123
Endocrine therapy	tamoxifen	0.169
	letrozole	0.794

[1]Wilcoxon test

Supplementary Figure 1

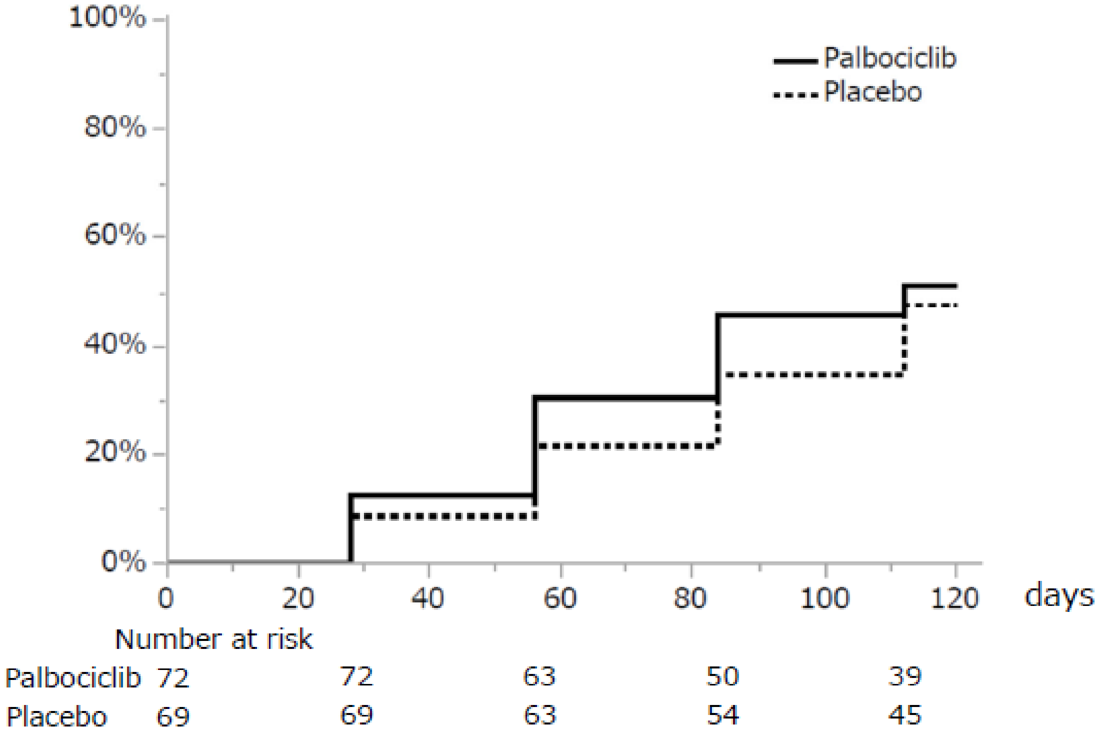

Figure legends

Supplementary Figure 1

Time to response by ultrasound in each arm

There was no statistical difference between two treatment arms (hazard ratio, 1.166; 95% confidence interval 0.729-1.865, p=0.484).
